# Oil of Sassafras for Neuralgia

**Published:** 1886-05

**Authors:** 


### Oil of Sassafras for Neuralgia.

This remedy is highly lauded by Dr. Thomas J. Miller in the *Virginia Medical Monthly*, who says that it may be given with more safety than the veratrum viride, and in the same diseases. In despondent, hysterical women, he has no doubt it would have a charming effect; and in fevers, and other cases of sanguineous engorgement, or of cerebral tendencies. He suggests that it might be a most eligible mode, whenever convenient, of giving it in a glass of soda water.

